# Functional Brain Imaging Synthesis Based on Image Decomposition and Kernel Modeling: Application to Neurodegenerative Diseases

**DOI:** 10.3389/fninf.2017.00065

**Published:** 2017-11-14

**Authors:** Francisco J. Martinez-Murcia, Juan M. Górriz, Javier Ramírez, Ignacio A. Illán, Fermín Segovia, Diego Castillo-Barnes, Diego Salas-Gonzalez

**Affiliations:** ^1^Signal Processing and Biomedical Application, Department of Signal Theory, Networking and Communication, University of Granada, Granada, Spain; ^2^Department of Scientific Computing, Florida State University, Tallahassee, FL, United States

**Keywords:** Alzheimer's Disease (AD), Parkinson's Disease (PD), Neuroimaging, Synthesis, density estimation, data augmentation, validation, evaluation

## Abstract

The rise of neuroimaging in research and clinical practice, together with the development of new machine learning techniques has strongly encouraged the Computer Aided Diagnosis (CAD) of different diseases and disorders. However, these algorithms are often tested in proprietary datasets to which the access is limited and, therefore, a direct comparison between CAD procedures is not possible. Furthermore, the sample size is often small for developing accurate machine learning methods. Multi-center initiatives are currently a very useful, although limited, tool in the recruitment of large populations and standardization of CAD evaluation. Conversely, we propose a brain image synthesis procedure intended to generate a new image set that share characteristics with an original one. Our system focuses on nuclear imaging modalities such as PET or SPECT brain images. We analyze the dataset by applying PCA to the original dataset, and then model the distribution of samples in the projected eigenbrain space using a Probability Density Function (PDF) estimator. Once the model has been built, we can generate new coordinates on the eigenbrain space belonging to the same class, which can be then projected back to the image space. The system has been evaluated on different functional neuroimaging datasets assessing the: resemblance of the synthetic images with the original ones, the differences between them, their generalization ability and the independence of the synthetic dataset with respect to the original. The synthetic images maintain the differences between groups found at the original dataset, with no significant differences when comparing them to real-world samples. Furthermore, they featured a similar performance and generalization capability to that of the original dataset. These results prove that these images are suitable for standardizing the evaluation of CAD pipelines, and providing data augmentation in machine learning systems -e.g. in deep learning-, or even to train future professionals at medical school.

## 1. Introduction

With the rise of neuroimaging in research and practice and the development of the machine learning paradigm, there has been an exponential trend in computer-aided methodologies (Frisoni et al., 2010; Martinez-Murcia et al., 2016; Rathore et al., 2017). Many Computer Aided Diagnosis (CAD) systems are being developed with application to structural and functional imaging in different diseases and disorders, such as Alzheimer's Disease (AD) (Stoeckel et al., 2004; Illán et al., 2011; Khedher et al., 2015) or Parkinson's Disease (PD) (Eckert and Edwards, 2007; Spetsieris et al., 2009; Segovia et al., 2016). However, these algorithms are usually tested in proprietary datasets to which the access is limited. This causes a series of problems in the evaluation of these systems, since a direct performance comparison is not enough to ensure validity. Furthermore, the false discovery rate (type I error) is often high in these studies due to a small sample size, significantly affecting reproducibility (Raudys and Jain, 1991; Poldrack et al., 2017).

A useful solution to perform direct comparison between different CADs is the use of multi-center datasets, and it has already been used in several challenges, such as the CAD Dementia challenge (Bron et al., 2015) or a recent Mild Cognitive Impairment (MCI) prediction challenge from Magnetic Resonance Imaging (MRI) at Kaggle (Sarica et al., 2016). Multi-center initiatives, such as the Alzheimer's Disease Neuroimaging Initiative (ADNI) (Weiner et al., 2012), the Autism Brain Imaging Data Exchange (ABIDE) (Di Martino et al., 2014) or the Parkinson's Progressions Markers Initiative (PPMI) (Marek et al., 2011) provide large image samples that reduce the type I error and ease a direct comparison between systems. However, this approach poses some fundamental limitations. First of all, although open data is gaining support in the community (Poldrack and Gorgolewski, 2014), access to these large datasets requires the approval of principal investigators or their teams, a common problem in neuroscience (Ferguson et al., 2014). Secondly, focusing on one, static dataset such as the aforementioned ADNI or PPMI might increase overfitting, reducing their generalization ability. And finally, inhomogeneities in scanner, population, or techniques might cause the apparition of false positives that are not related to the signal (Pearlson, 2009; Van Horn and Toga, 2009).

For its part, synthetic datasets have been widely used in the pattern recognition community in different ways. Synthetic images have all the information about how they have been generated, and so can be used as a ground truth for automatic systems (Black et al., 2003; Ros et al., 2016; Varol et al., 2017). They can also significantly increase the sample size in a procedure commonly known as data augmentation, via analysis-synthesis (Cui et al., 2004) or performing deformations (Krizhevsky et al., 2012). This is a key feature that allowed a faster development of deep learning approaches that need huge amounts of data to build models.

In neuroimaging, some of the previous approaches have already percolated research practices. Recent machine learning challenges such as the aforementioned Kaggle MCI-MRI (Sarica et al., 2016), included synthesized morphological data to prevent overfitting and *ad-hoc* model training. There are initiatives to build ground-truth phantoms for developing and studying new scanner technologies (Jan et al., 2004; Segars et al., 2010; Stute et al., 2011), to study generative models of functional activation in fMRI (Yarkoni et al., 2011; Erhardt et al., 2012) or to evaluate segmentation procedures such as the BrainWeb initiative (Kwan et al., 1999). However, brain image synthesis algorithms are rarely used for standardization or data augmentation, with few examples of spatial transformations and deformations in MRI (Zhu et al., 1994; Xue et al., 2006) or SPECT-DaTSCAN (Ronneberger et al., 2015; Martinez-Murcia et al., 2017).

**Figure 1:**
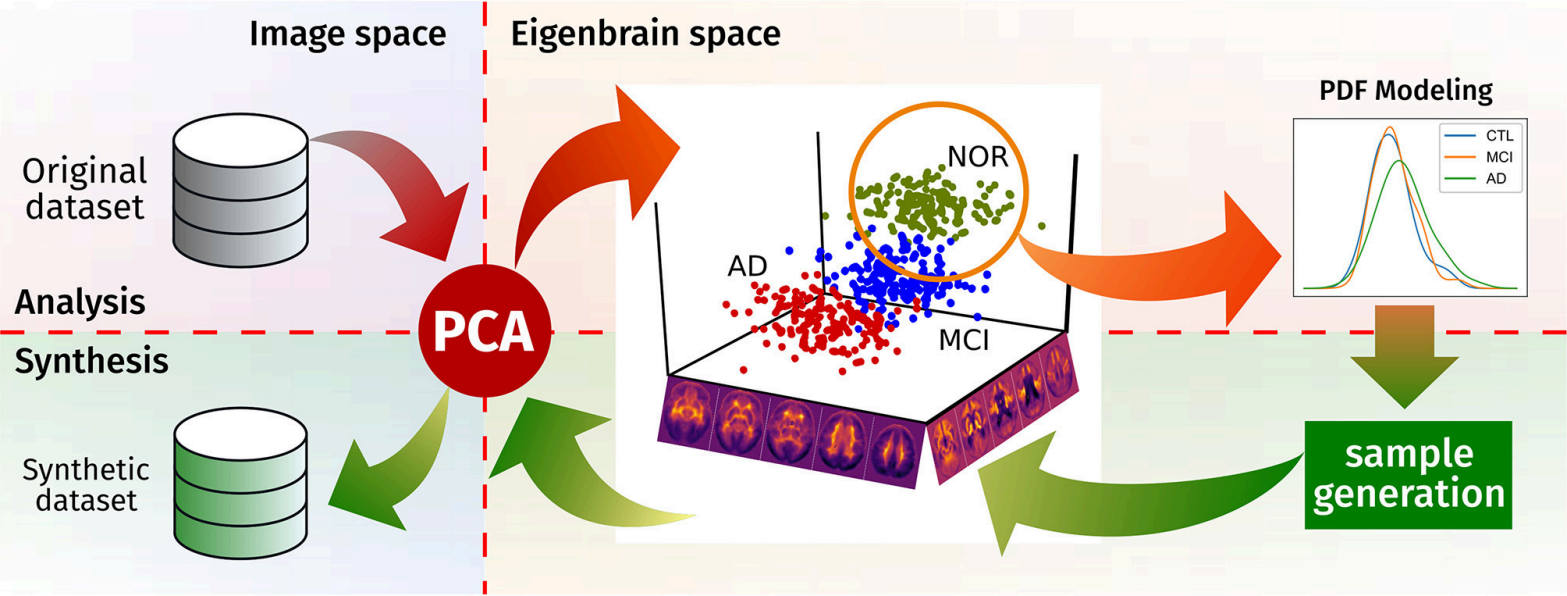
Schema of the proposed synthesis procedure.

In this work, we provide a novel brain synthesis technique (see Figure 1) to address the data augmentation and the ground truth problems in neuroimaging by generating a new set of images that share characteristics with a known dataset. Our system performs an analysis of that existing database and extracts a common orthogonal “eigenbrain” basis of a multidimensional space where the different subjects are represented as a coordinate vector, using Principal Component Analysis (PCA), as in many neuroimaging analysis and synthesis papers (Zhu et al., 1994; Markiewicz et al., 2009; Illán et al., 2011; Khedher et al., 2015). In contrast to them, our methodology creates per-class/modality models of the statistical distribution of the coordinates in this space. Together with a random sampling based on the Cumulative Density Function (CDF), it allows us to draw new uncorrelated coordinates from each model, thus setting the ground truth. This is of special importance where class clusters overlap due to the progression of the disease (e.g., MCI and AD), providing samples for standardizing the evaluation of CAD systems.

We have tested this generation approach against two widely used datasets: a Positron Emission Tomography (PET) dataset from the ADNI initiative, and a Single Photon Emission Computed Tomography (SPECT) dataset using the drug DaTSCAN, from the Parkinson's Progression Markers Initiative (PPMI). Our purpose is to assess that the intrinsic properties of each class in these modalities is kept, while providing uncorrelated images that can effectively predict real-world samples, thus reducing bias in the evaluation of new CAD methods. Additionally, the educational use of synthetic images to train future professionals is an inviting possibility yet to explore.

## 2. Methodology

### 2.1. Databases and preprocessing

We tested our synthesis methodology on two large datasets comprising AD and PD:

#### 2.1.1. Alzheimer's disease neuroimaging initiative (ADNI)

The first dataset used in the preparation of this article were obtained from the Alzheimer's Disease Neuroimaging Initiative (ADNI) (adni.loni.usc.edu). The ADNI was launched in 2003 as a public-private partnership, led by Principal Investigator Michael W. Weiner, MD. The primary goal of ADNI has been to test whether serial MRI, PET, other biological markers, and clinical and neuropsychological assessment can be combined to measure the progression of Mild Cognitive Impairment (MCI) and Alzheimer's Disease (AD). For up-to-date information on the data used, the recruitment process and the image properties, see www.adni-info.org and Weiner et al. (2012).

For this work, we focus on the ^18^*F*-FDG PET images available. This radiopharmaceutical is a glucose analog, and its distribution of the brain can be used to trace glucose metabolism, and by extension, brain function. The ADNI PET subset here contains *N* = 403 images: 95 images from individuals affected by AD, 207 images from MCI subjects and 101 images from Controls (NOR).

#### 2.1.2. Parkinson's progression markers initiative (PPMI)

Another large multi-center dataset used in the preparation of this article was obtained from the Parkinson's Progression Markers Initiative (PPMI) database (www.ppmi-info.org/data). For up-to-date information on the study, visit www.ppmi-info.org and (The Parkinson Progression Marker Initiative, 2010; Marek et al., 2011). The dataset contains SPECT images obtained using the radiopharmaceutical ioflupane, also known as its trade name DaTSCAN, which binds to the dopamine transporters at the striatum. This allows to quantify the dopaminergic deficit associated to Parkinson's Disease (PD). In this study we use 269 DaTSCAN images belonging to 111 NOR and 158 subjects affected by PD.

#### 2.1.3. Database preprocessing

The aforementioned datasets have been preprocessed to account for spatial and intensity differences. A spatial normalization, also known as registration, has been applied to ensure that the same MNI coordinate corresponds to the same spatial position inside the brain. We have used the SPM8 software (Friston et al., 2007) to perform this task, using either the included PET template (for the ADNI dataset) and a custom template (Salas-Gonzalez et al., 2015) for the PPMI and VDLN.

Later, intensity normalization was applied to ensure that a direct comparison of the image function encoded in the voxel intensities (dopamine transporters density in DaTSCAN and glucose metabolism in FDG-PET) is possible. This corrects the individual differences (e.g., drug uptake, exposition time, etc.) that affect these values, and is also key since the subsequent analysis needs directly comparable values to quantify variance. We have applied a normalization to the maximum strategy (Saxena et al., 1998) in the form ${\text{I}}^{\prime }=\text{I}/I_{n}$, where the image intensity **I** is divided by *I*_*n*_, the average of the top 3% intensities. This normalization was proven very useful in Illán et al. (2011, 2012) and Martínez-Murcia et al. (2012, 2014).

### 2.2. Principal component analysis (PCA)

Principal Component Analysis (PCA) is used to establish a common reference, or basis, to generate new images. It is very extended in analysis and feature extraction in neuroimaging (Markiewicz et al., 2009; Illán et al., 2011; Khedher et al., 2015), and also was used with the same purpose of obtaining a common neuroimaging reference in Zhu et al. (1994).

Intuitively, PCA defines a new space where the first spatial direction is defined so that it explains the maximum variance in the data. The subsequent directions will try to explain the remaining variance in decreasing order. All these directions, or components, are meant to be uncorrelated. This way, the maximum information about the data is contained in the first components, and the remaining can be considered noise.

Mathematically (Brown, 2009), PCA works as an orthogonal transformation that maps a correlated set of observations **X** (in this work, our set of zero-rated images, of size *K* × *N* containing *K* images of length *N*) into a set of uncorrelated data **S** that contains *K* vectors in a *M*-dimensional space, defined by **W**, a vector whose *M* columns contains the basis of the new space. That way, the mapping is obtained by:

(1) $$\begin{array}{r} \text{S}=\text{X}\text{W} \end{array}$$

where the columns of **W** contain the eigen-values of **X**^*T*^**X**, the empirical covariance matrix of **X**.

This is done, ideally, by obtaining the eigen-value decomposition of the empirical covariance matrix of the data. A very extended and fast way of computing **W**, the matrix of eigenvectors, also known as “eigenbrains” in the neuroscience literature (Illán et al., 2011), is via the Singular Value Decomposition (SVD) of **X**:

(2) $$\begin{array}{r} \text{X}=\text{U}\Sigma {\text{V}}^{*} \end{array}$$

where **U** is a *K* × *K* orthogonal matrix, **Σ** is a *K* × *M* non-negative real diagonal matrix, and the *M* × *M* unitary matrix V^*^ denotes the conjugate transpose of the *M* × *M* unitary matrix **V**. Using this decomposition, we can rewrite Equation (1) as:

(3) $$\begin{array}{r} \text{S}=\text{X}\text{W}=\text{U}\Sigma \end{array}$$

Given that the conjugate transpose of a unitary matrix is its inverse, the matrix **W** is equivalent to **V**. A truncated version of the decomposition can also be performed, by retaining the first *L* components (ranked by their eigen-values):

(4) $$\begin{array}{r} {\text{S}}_{L}=\text{X}{\text{W}}_{L} \end{array}$$

where **S**_*L*_ is the truncated estimate of size *K* × *L*, and **W**_*L*_ contains only the *L* first columns of **W**. Since PCA does not account for random noise in its model, the noise is included as different components. Therefore, a choice for *L* can eliminate random noise and increase the signal to noise ratio of our model. In this work, when not stated, we will use the first *L* = *K* components (where *K* is the number of samples in the dataset).

### 2.3. Density estimation

Once the images have been projected to the eigenbrain space, we want to generate new samples in this new space, in order to synthesize new images. To do so, we assume that the coordinates of the subjects of a certain class in the eigenbrain space are different realizations of a random process with a given Probability Density Function (PDF).

In order to estimate the PDF of the process that generates the coordinates of each class, we use two different density estimation procedures: a multivariate approach under the assumption of a normal distribution and a per-component estimation of density using the empirical Kernel Density Estimation.

#### 2.3.1. Multivariate normal distribution (MVN)

The Multivariate Normal Distribution (MVN, also known as Multivariate Gaussian Distribution) is a generalization of the random normal distribution to *n* dimensions.

Let us note **S**^*c*^ the matrix containing only the coordinates in the eigenbrain space of the *K*_*c*_ individuals belonging to class *c*. We can estimate its multivariate PDF ${\hat{f}}_{mvn}^{c}\left(\text{x}\right)$ by computing the class mean **μ**^*c*^ and its class covariance matrix **Σ**^*c*^. The estimation of **Σ**^*c*^ is performed via shrinkage, which consists in reducing the ratio between the smallest and the largest eigen-value of the empirical covariance matrix using a shrinkage parameter α. In this work we used the method proposed in Ledoit and Wolf (2004) to estimate an optimum α that minimizes the Mean Squared Error between the estimated and the real covariance matrix. The multivariate PDF for class *c* would be:

(5) $$\begin{array}{r} {\hat{f}}_{mvn}^{c}\left(\text{x}\right)=\frac{1}{{\left(2\pi \right)}^{K_{c}/2}|\Sigma^{c}{|}^{1/2}}\exp\left(\frac{{\left(\text{x}-\mu^{c}\right)}^{T}\Sigma_{c}^{-1}\left(\text{x}-\mu^{c}\right)}{2}\right) \end{array}$$

#### 2.3.2. Kernel density estimation (KDE)

Kernel Density Estimation (KDE) is an increasingly used method to estimate the PDF of a set of data (Botev et al., 2010; Simonoff, 2012). In this case, we perform a per-component estimation of the PDF. This approach disregards conditional probabilities between components and uses each component's modeling as independent. While this is not theoretically accurate, the components extracted in PCA are uncorrelated by definition, and in practice the conditional terms are very small.

On the other hand, the per-component KDE is less prone to overfitting by disregarding these constraints. Additionally, the KDE can empirically account for heavy-tailed distributions that are sometimes more common in pathological models (Salas-Gonzalez et al., 2012), which might make this model more suitable.

An estimation of the PDF of the *l*th coordinate of class *c* is defined using KDE as:

(6) $$\begin{array}{r} {\hat{f}}_{kde}^{l,c}\left(x\right)=\frac{1}{K_{c}}\overset{K_{c}}{\underset{i=1}{\sum }}G_{h}\left(x-{\text{S}}_{l}^{i,c}\right)=\frac{1}{K_{c}h}\overset{K_{c}}{\underset{i=1}{\sum }}G\left(\frac{x-{\text{S}}_{l}^{i,c}}{h}\right), \end{array}$$

where *i* = 1, …*K*_*c*_, with *K*_*c*_ the number of subjects belonging to class *c*, *h* is the bandwidth and ${\text{S}}_{l}^{i,c}$ contains the *l*th coordinate of the *i*th subject of class *c*. The kernel *G*(*x*) is a function of ℝ^*n*^ that must define a probability:

(7) $$\begin{array}{r} {\int \cdot \cdot \cdot \int }_{{\mathbb{R}}^{n}}G\left(\text{x}\right)d\text{x}=1 \end{array}$$

It also must be centered:

(8) $$\begin{array}{r} {\int \cdot \cdot \cdot \int }_{{\mathbb{R}}^{n}}\text{x}G\left(\text{x}\right)d\text{x}=0 \end{array}$$

and its covariance matrix must be close to identity:

(9) $$\begin{array}{r} \forall \text{u}\in {\mathbb{R}}^{n},||\text{u}||=1\;\int_{\mathbb{R}}t^{2}G\left(t\text{u}\right)dt\approx 1 \end{array}$$

In this work, we use a gaussian kernel $G\left(x\right)=1/\left(2\pi \right)\exp\left(-\frac{1}{2}x^{2}\right)$ for the estimate. Estimation of the bandwidth *h* is performed using the diffusion approximation proposed by Botev et al. (2010).

### 2.4. Brain image synthesis

After estimating the empirical PDF of the coordinates, we aim to generate a new set of coordinates for class *c* $\hat{S^{c}}$ that match the distribution of the originals. To do so, we compute the Cumulative Distribution Function (CDF) from the PDF that we estimated previously as:

(10) $$\begin{array}{r} F\left(x\right)=\int_{-\infty }^{x}f\left(t\right)dt \end{array}$$

Afterwards, we can use a random number generator to provide uniformly distributed random numbers in the interval [0, 1]. These numbers are in the range of the CDF (from 0 to 1), and therefore we can consider them as *F*(*x*), from which we could obtain the value *x*. In practice, we perform a numerical approximation to the problem, in which we calculate the full CDF in a wide range of *x*, and then interpolate the value of *x* using the generated *F*(*x*) as query point.

This procedure is repeated for all coordinates *i* = 1, …*K* as many times as the number of subjects of class *c* that we want to synthesize. Then, the new set of images can be reconstructed using the eigenbrain basis **W** and the new matrix of scores $\hat{S^{c}}$.

(11) $$\begin{array}{r} {\hat{\text{X}}}^{c}=\hat{S^{c}}{\text{W}}^{-1} \end{array}$$

The synthesis procedure defined here is available via the **brainSimulator** python package at https://github.com/SiPBA/brainSimulator, under the GPL-3+ license.

### 2.5. Validation

Validation of a synthetic dataset is still a matter of discussion. It depends heavily on the specific application of the synthesis. For example, for synthetic phantoms (Jan et al., 2004; Segars et al., 2010; Stute et al., 2011) and automatic segmentation methods (Ma et al., 1993; Kwan et al., 1999), visual inspection was used. Additionally, some studies performed measures of accuracy of segmentation, but that is not our case. Other studies applied spatial deformations for data augmentation (Zhu et al., 1994; Xue et al., 2006; Ronneberger et al., 2015; Martinez-Murcia et al., 2017), mostly without validating the deformed images.

The main purpose of this work is to generate images that could have been drawn from the same population of a given dataset, sharing relevant characteristics and, at the same time, being independent from the existing samples. Assessing this is not trivial. Therefore, we use two kinds of analyses:

- The well-known **Statistical Parametric Mapping** analysis (SPM) (Friston et al., 2007), to obtain a visual identification of the differences and similarities between classes and datasets. In particular, mass-univariate two-sample *t*-maps were obtained using the SPM12 software, using Family-wise error (FWE) correction *t*-threshold for a *p* < 0.05, with no masking applied.
- A classification analysis using **Voxel as Features** (VAF) (Stoeckel et al., 2004), which yields classification performance on different experimental setups. We cross-validate (10-fold) a Support Vector Machine Classifier (SVC) with linear kernel where the voxel intensities are used as features. To estimate the SVM regularization parameter (*C*), we perform a grid search in an inner 5-fold cross-validation loop on the training set. The following performance values are provided: accuracy (acc), sensitivity (sens), specificity (spec), and their standard deviations (SD).

These analyses will be applied to evaluate the:

- **E1: Similarity and generalization ability** of the synthetic datasets via three different approaches:
  - **E1.1**. Assessing the **differences between classes** in both the original and the synthetic images using SPM and classification analysis. Similar performance results and SPM significant areas is expected if the original datasets are accurately modeled. We also study here the dependence of the synthetic images on the model parameters *L* (number of components) and *N* (number of generated subjects).
  - **E1.2**. Assessing the **differences between original and synthetic datasets**, using both SPM and classification analysis. If we assume that the synthetic datasets are a new sampling of the population from which the original datasets were drawn, there should be no significant differences between them.
  - **E1.3**. Evaluating the **predictive power of the synthetic images** on the original datasets. In this case, we use the synthetic images as a training set and test de trained model on real-world samples.
- **E2: Independence of the synthetic images**. To assess how much the synthesis procedure depends on the original dataset we choose a strategy based on the resubstitution error (Neto and Dougherty, 2015). Resubstitution (using the training set as test set) estimates the performance loss in the training. We will obtain the resubstitution error of the original training set and a synthetic dataset derived from that original training set. If this performance loss is similar to the performance loss in the original test set (E1.1), this means that we can consider that our synthesis algorithm produces images independent from the original set.

## 3. Results

### 3.1. E1: similarity and generalization ability of the synthetic datasets

#### 3.1.1. E1.1: differences between classes

The first approach we used to verify whether the synthetic images are similar to their original counterparts is to quantify and localize differences between classes in both the original and the synthetic datasets. We assess this similarity using both a map of the statistical differences between classes in all these datasets (SPM) and the classification performance when using the original and the synthetic images.

**Figure 2:**
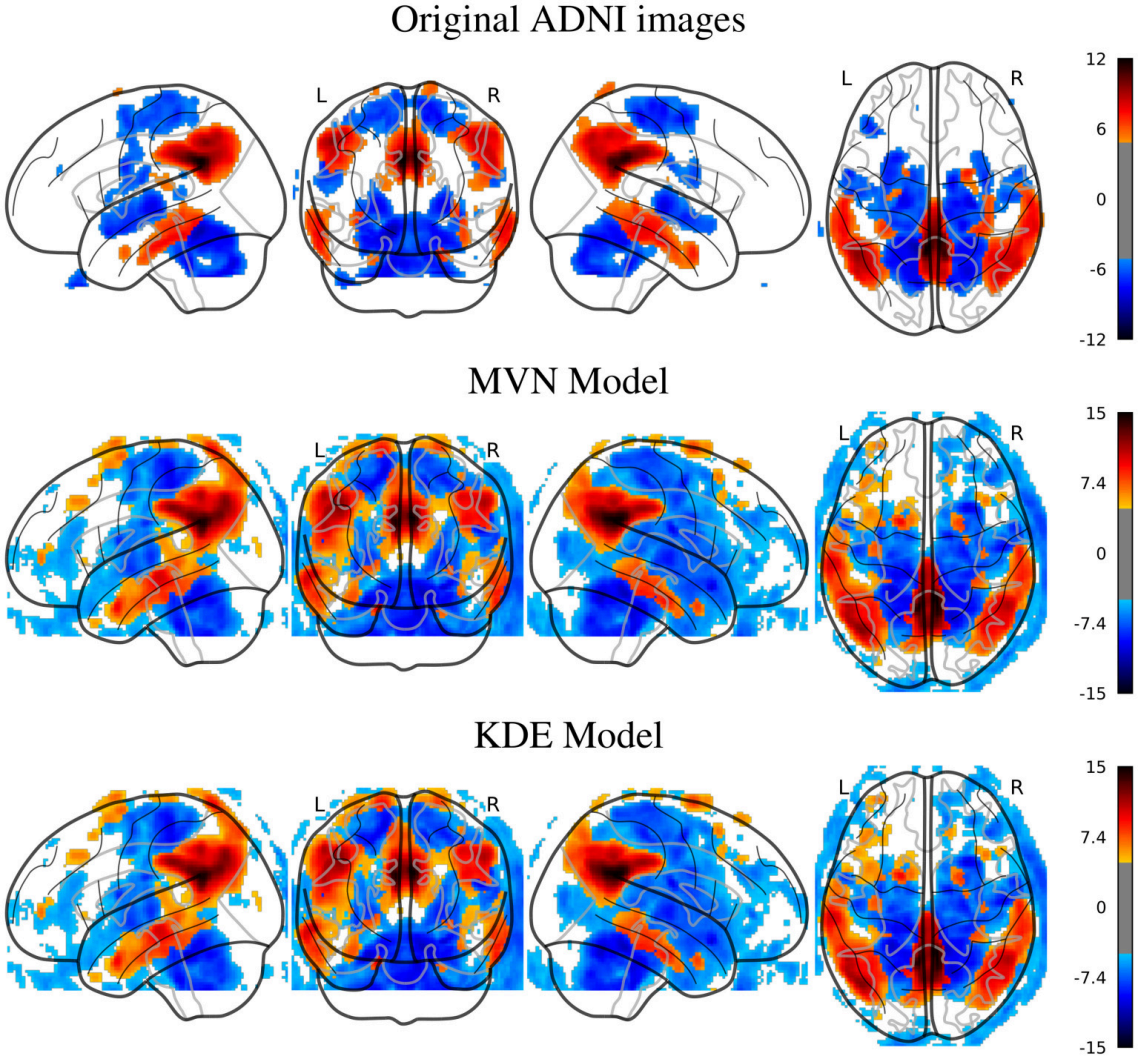
SPM analysis of the ADNI dataset (AD vs. NOR), Family-Wise Error (FWE) corrected, with *p* = 0.05, for the original and the synthetic images.

In Figure 2, we can look at the differences (*t*-maps, FWE corrected, *p* < 0.05) between AD and NOR images with the original, and two synthesized datasets with 200 samples per class, using either MVN or KDE modeling. In these maps, the differences are located in similar places in both synthesized databases, that are as well, although less intense, represented in the SPM analysis of the original dataset. The aforementioned regions such as the precuneus, angular, mid-temporal lobe, hippocampus, amygdala, among others, are represented in both the original and synthetic datasets, with a special mention of the cingulum, which also is the main difference in the MCI vs. NOR scenario (see Supplementary Material).

**Figure 3:**
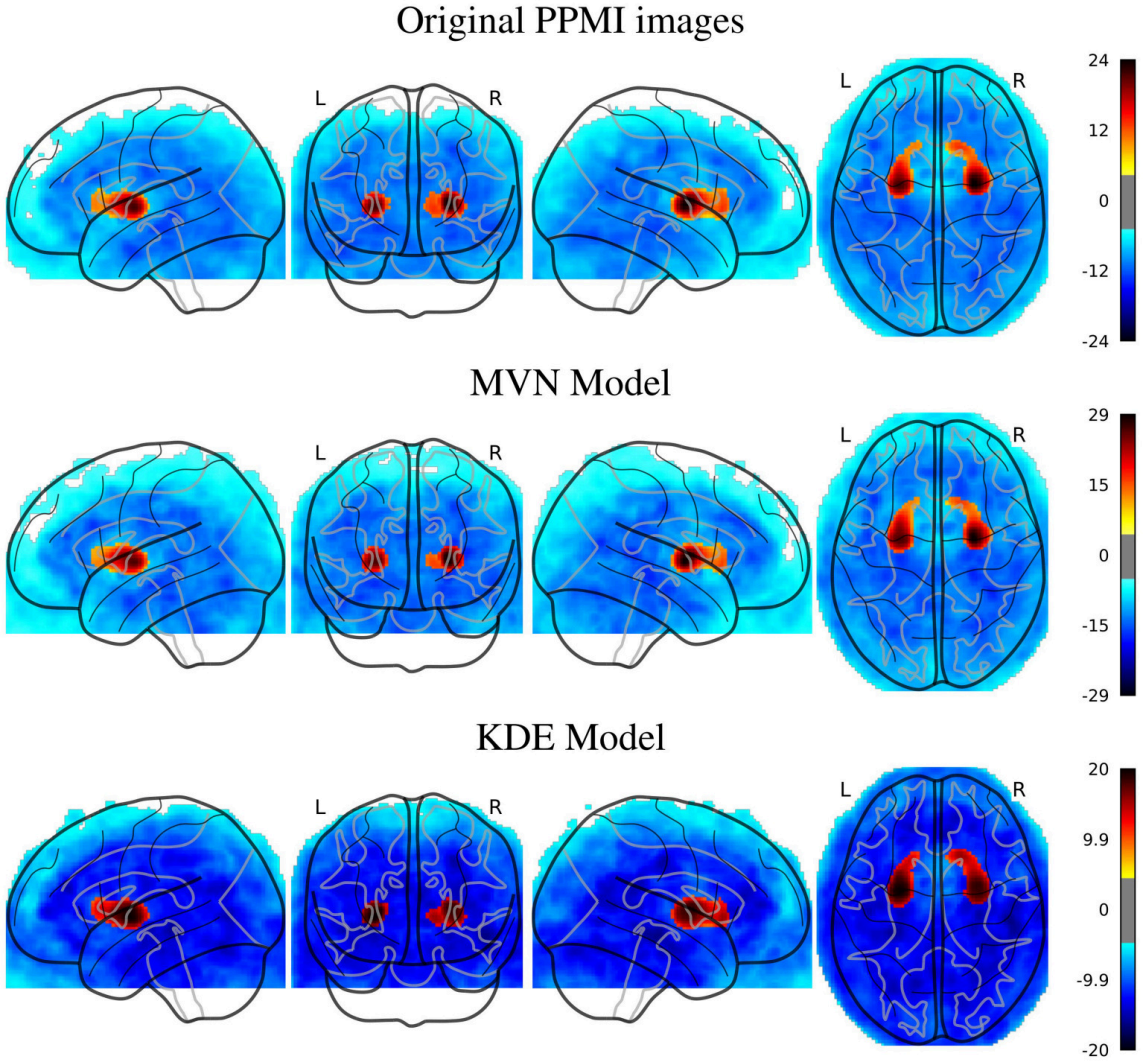
SPM analysis of the PPMI dataset (PD vs. NOR), Family-Wise Error (FWE) corrected, with *p* = 0.05, for the original and the synthetic images.

In the PPMI dataset, the SPM analysis revealed (Figure 3) that the main differences are located in the posterior part of the striatum, specifically at the posterior part of the putamen and globus pallidus. This behavior is consistent in both original and synthesized images, although with more statistical significance in the case of the MVN model, and a more homogeneous distribution of the negative differences under the KDE model.

The choice of these two datasets was not random. They represent two extremes of the nuclear imaging spectrum: a very specific radiotracer (DaTSCAN) in two distinguishable states of a disease (PPMI) and a general radiopharmaceutical (FDG PET) in a progressive neurodegenerative disorder with very overlapping classes (ADNI). The separation between AD and NOR should be, therefore, higher than between the intermediate MCI and the two better-defined classes. However, even the AD and NOR classes overlap in the original dataset, perhaps due to a lower specificity of the biomarker (glucose metabolism) and noise in the diagnostic tests used to label the patients (Chapman et al., 2016).

A more descriptive analysis can be performed via the VAF performance of the original and synthetic datasets. These results can be used to quantify the impact of some model parameters (for example, the number of subjects generated *N* or the number of components *L* used in the model) on the differences between classes. To do so, we first establish a baseline: the VAF performance of the original dataset, that can be checked at Table 1.

**Table 1:** Original VAF performance of the two datasets, including MCI scenario in ADNI.

**Database**	**Scenario**	**acc [SD]**	**sens [SD]**	**spec [SD]**
PET ADNI	AD vs. NOR	0.882 [0.012]	0.865 [0.091]	0.901 [0.118]
	MCI vs. NOR	0.698 [0.042]	0.791 [0.064]	0.504 [0.179]
	MCI vs. AD	0.702 [0.117]	0.444 [0.219]	0.822 [0.258]
DAT PPMI	PD vs. NOR	0.923 [0.057]	0.929 [0.090]	0.918 [0.088]

Now, two of the model parameters, *L* and *N* have been varied to explore their impact on the synthetic images. First, we vary *L*, the number of components used to synthesize images. It is logic to assume that a small number of components will be insufficient to acknowledge all variance in the original dataset, therefore producing low-quality images. On the other hand, a large number of components could lead to overfitting, especially in the MVN model, which takes into account not only the component scores' distribution, but also the relations between components.

**Figure 4:**
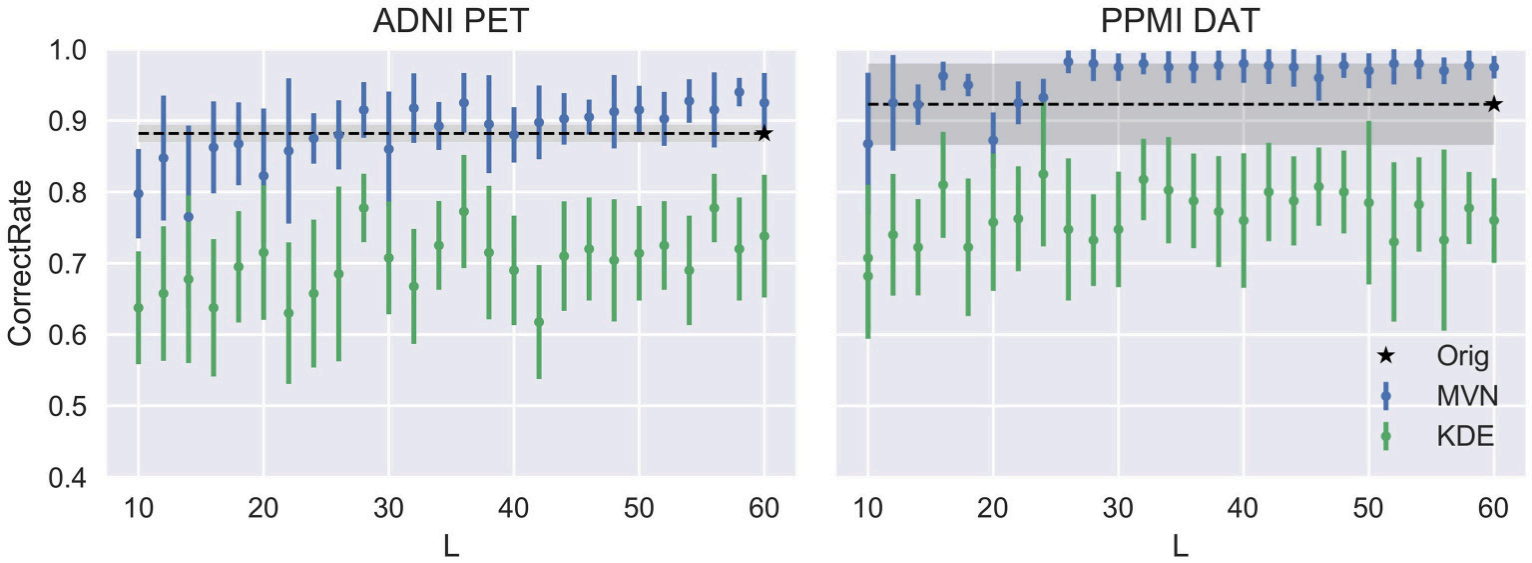
Evolution of the accuracy when varying the number of components *L*.

This evolution is assessed in Figure 4, compared to the VAF performance of the original dataset (black dashed line) and standard deviation (shadowed area). There, we can check that our assumptions were accurate. Small *L*s lead to inaccurate models where the VAF performance is low. On the other hand, larger *L*s have a stronger impact on the MVN modeling, while the KDE performance remains almost unaltered after a certain *L*-value. This behavior might be an indication that the MVN approach converges faster to an optimum modeling (approximate error 0.15 in ADNI and 0.08 in PPMI), and afterwards starts to produce more concentrated data clusters in the eigenbrain space due to the restrictions imposed by its *L*-dimensional nature. The larger the number of components *L* is, the more concentrated the data clusters might be, a possible indication of overfitting. We can also observe that the MVN surpasses the original VAF performance with a smaller *L*, which points to the fact that the variability in DaTSCAN imaging is far smaller than in PET, and therefore, less components are needed for an accurate model.

We can assume that the optimum model uses the *L* that produces the more detailed images while maintaining a similar performance to the original dataset. In this work we propose to choose the highest *L* whose average performance remains within 1 standard deviation of the original dataset performance. In our case, it would be *L* = 40 for the FDG-PET and *L* = 24 for DaTSCAN. Now, by fixing these two *L*s, we will analyze how the VAF performance changes when increasing the number of synthetic images. We may assume that increasing *N* would reduce progressively the standard deviation of the performance estimates, converging toward the original VAF performance.

**Figure 5:**
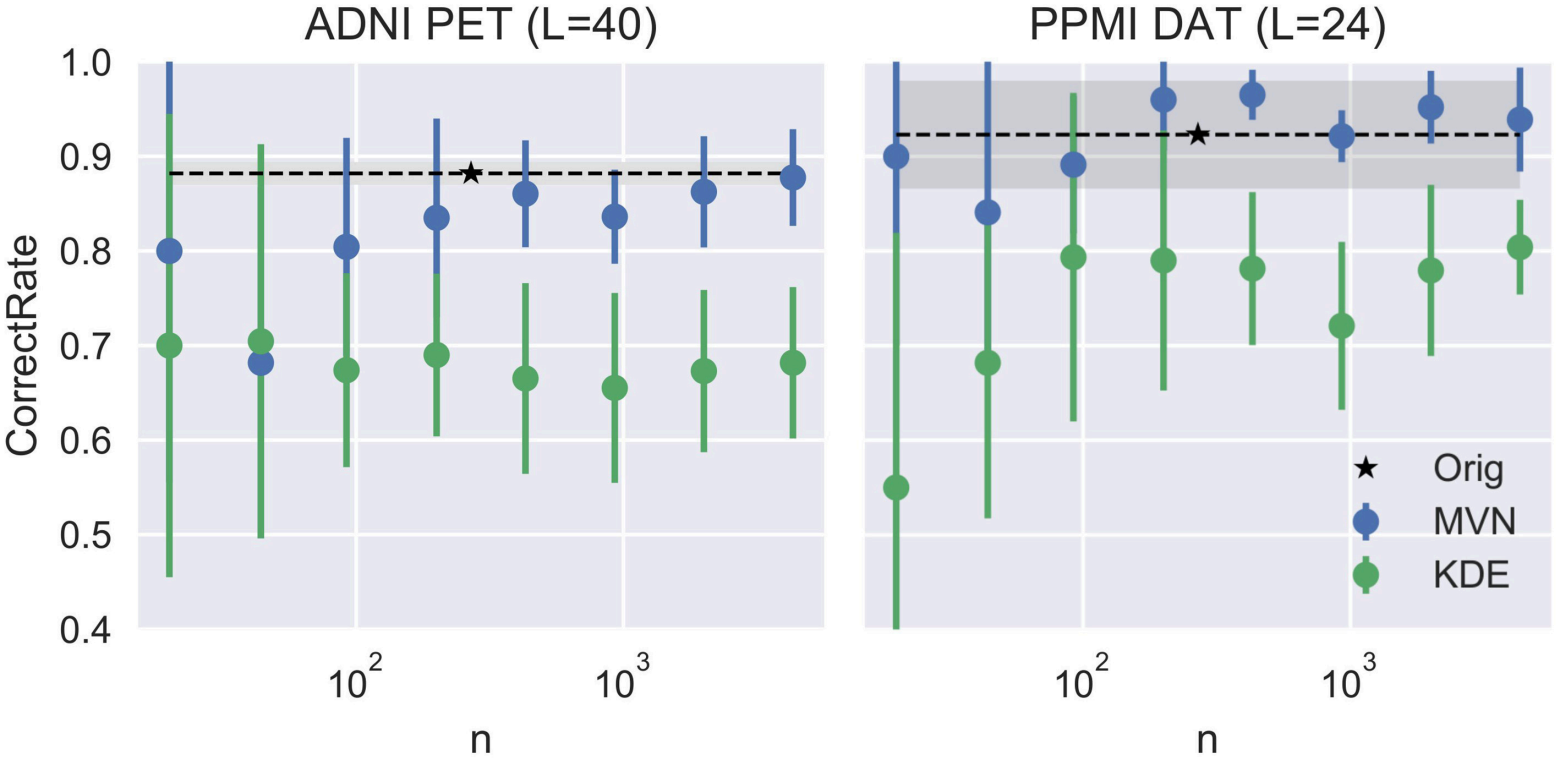
Evolution of the accuracy when varying the number of synthetic images *N*.

Figure 5 shows that, while the VAF performance of the MVN modeling increases and reduces its variance, eventually reaching the original performance, the KDE estimation hardly varies after a decent sample size (100 subjects, 50 subjects per class) has been reached.

### 3.2. E1.2: differences between original and synthetic images

It is now important to ensure that the synthetic images statistically belong to the same population from which the original images were drawn. To do so, we perform a SPM analysis (massive univariate *t*-test with FWE corrected with *p* = 0.05) that compares the original datasets to:

- KDE-synthetic (with maximum *L*) datasets.
- KDE-synthetic (with *L* = 40/24 for ADNI/PPMI) datasets.
- MVN-synthetic (with maximum *L*) datasets.
- MVN-synthetic (with *L* = 40/24 for ADNI/PPMI) datasets.

Therefore, eight analyses were performed (four per dataset). None of these analyses yielded significant (*p* < 0.05, FWE) differences between the two populations, regardless of the model type (MVN or KDE) or database. Therefore, the group differences between original and synthetic images were relatively sparse and of small effect sizes. This might indicate that both the original and the synthetic images belong to the same population.

### 3.3. E1.3: generalization ability of the synthetic images

**Figure 6:**
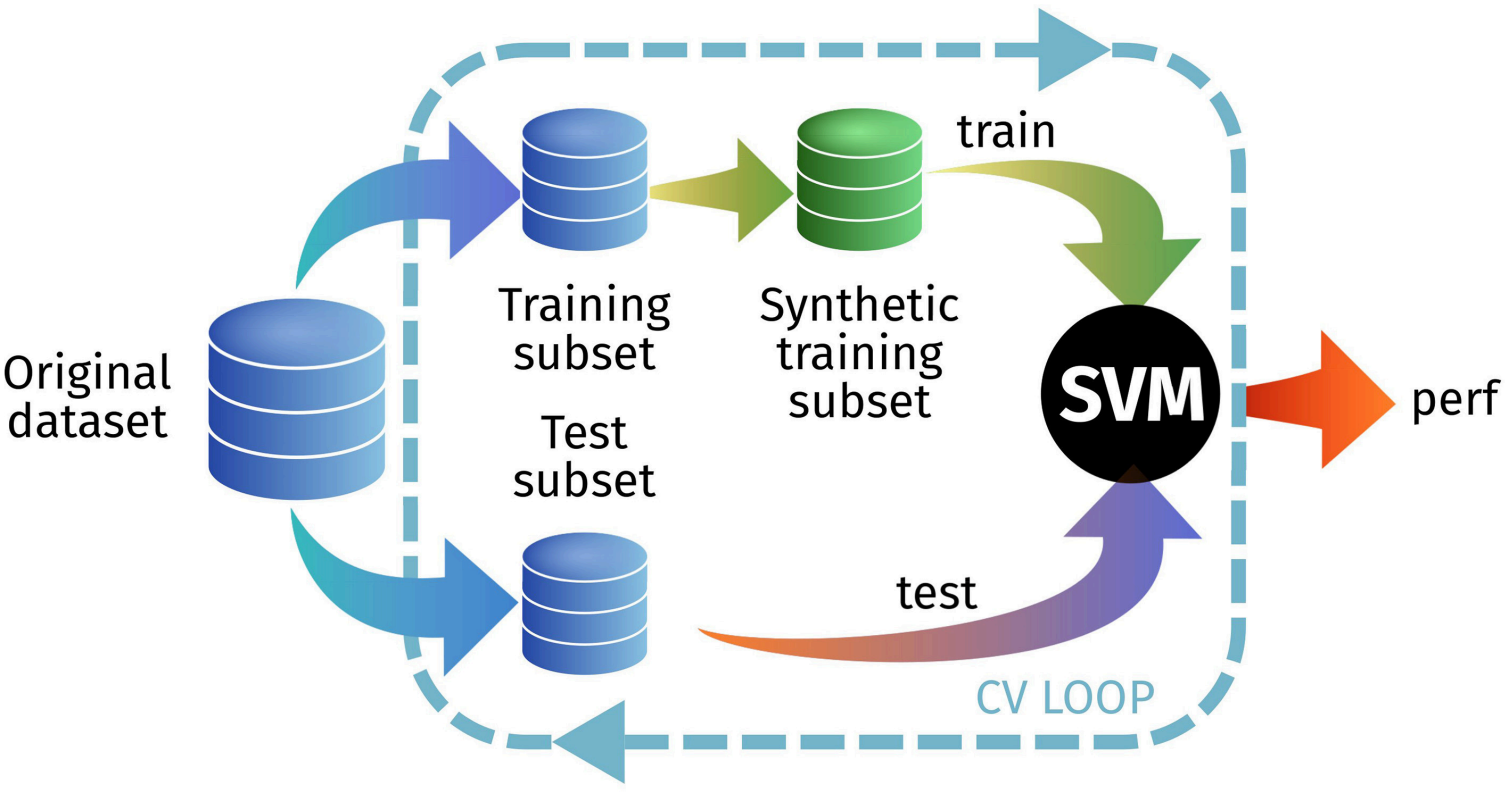
Outline of the experimental setup for E1.3 to test the generalization ability of the synthetic images.

In the first experiment, we test how a model trained with synthetic images is able to predict real-world images. We do this by generating a synthetic training set from the original training set within the cross-validation loop, as seen in Figure 6. We tested this approach on the PET ADNI and the DAT PPMI datasets, using all PDF estimators and different *L*-values. In each cross-validation iteration we synthesize a new set with 200 samples per class. Results are shown at Table 2.

**Table 2:** Performance of E1.3, demonstrating the predictive ability of the synthetic images over the real dataset.

**Database**	**Est**.	***L***	**Scenario**	**acc [SD]**	**sens [SD]**	**spec [SD]**
PET ADNI	MVN	403	AD vs. NOR	0.852 [0.078]	0.804 [0.151]	0.900 [0.137]
			MCI vs. NOR	0.688 [0.067]	0.743 [0.108]	0.572 [0.169]
			MCI vs. AD	0.675 [0.121]	0.468 [0.192]	0.774 [0.224]
		40	AD vs. NOR	0.790 [0.084]	0.813 [0.165]	0.770 [0.207]
			MCI vs. NOR	0.496 [0.139]	0.374 [0.359]	0.750 [0.392]
			MCI vs. AD	0.622 [0.152]	0.563 [0.298]	0.651 [0.320]
	KDE	403	AD vs. NOR	0.770 [0.114]	0.747 [0.153]	0.790 [0.169]
			MCI vs. NOR	0.642 [0.044]	0.726 [0.098]	0.472 [0.188]
			MCI vs. AD	0.672 [0.081]	0.484 [0.142]	0.760 [0.187]
		40	AD vs. NOR	0.622 [0.114]	0.784 [0.290]	0.459 [0.394]
			MCI vs. NOR	0.509 [0.152]	0.467 [0.434]	0.599 [0.450]
			MCI vs. AD	0.576 [0.170]	0.522 [0.363]	0.602 [0.390]
DAT PPMI	MVN	268	PD vs. NOR	0.948 [0.041]	0.948 [0.055]	0.946 [0.064]
		24	PD vs. NOR	0.925 [0.047]	0.910 [0.079]	0.945 [0.083]
	KDE	268	PD vs. NOR	0.914 [0.082]	0.916 [0.113]	0.909 [0.121]
		24	PD vs. NOR	0.833 [0.146]	0.834 [0.292]	0.819 [0.248]

The prediction accuracy, sensitivity and specificity are higher when using the MVN estimator than with the KDE in both ADNI and PPMI. The MVN modeling performance is also closer to the original baseline performance (see previous section), especially when using the suggested *L* = 40 for AD and *L* = 24 for PD. The KDE performance, however, degrades significantly when reducing the number of components.

Regarding the PET ADNI dataset, we tested three different scenarios: AD vs. NOR, MCI vs. NOR, and MCI vs. AD. The higher performance is obtained in the AD vs. NOR, but when including MCI subjects, the results vary. In the case of the MVN estimator, the MCI vs. NOR scenario performs slightly better than the MCI vs. AD. On the other hand, when using the KDE, MCI vs. AD obtains better results than MCI vs. NOR. However, the big difference among them is that, whereas the MVN estimator achieves similar performance to the baseline, the KDE-synthetic images lead to smaller predictive power.

### 3.4. E2: dependence on the original images

In experiment 2, we will assess the dependence of the synthetic images on the original datasets, using the resubstitution error (Neto and Dougherty, 2015). A classifier trained and tested on the same set (resubstitution) usually has a low error rate (resubstitution error or training error). It is generally assumed that the generalization error is the test error, or the error achieved by a test set, different from the training set.

**Figure 7:**
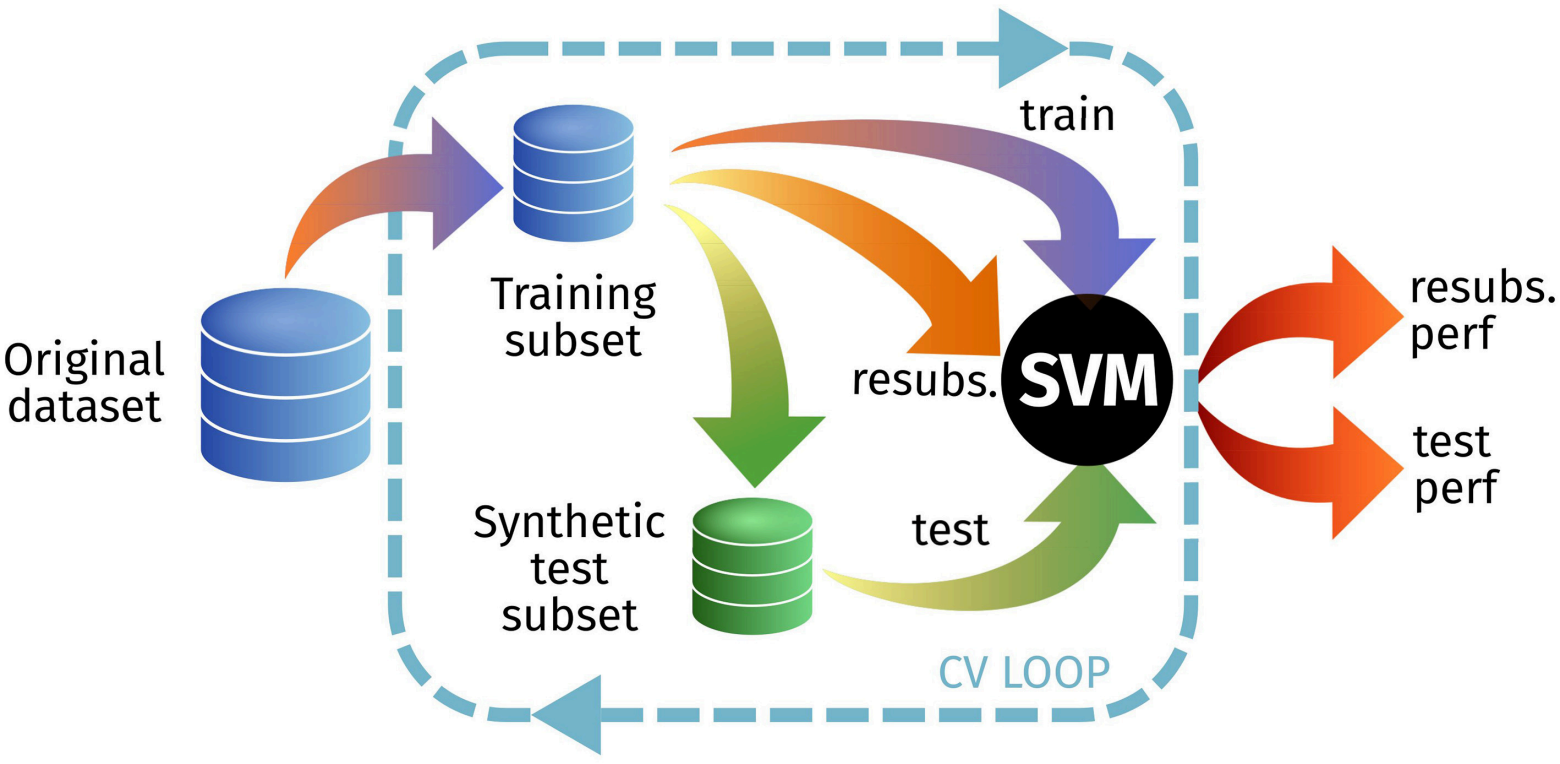
Outline of the experimental setup for E2 to test the resubstitution and prediction accuracies.

In this experiment, we first estimate the resubstitution accuracy (1− resubstitution error) testing on the original training set, and then use that same set to synthesize (at different *L*s) a new test set (Figure 7). Depending on the performance loss, that could imply realistic images—different from the training set—or images that are almost identical to the training set–overfitting. The results for this experiment are shown at Table 3.

**Table 3:** Performance of E2, showing the resubstitution error (resubs.) and the test error of synthetic test sets with different *L*s.

**Database**	**Scenario**	**Test (model**, ***L*****)**		**acc [SD]**	**sens [SD]**	**spec [SD]**
PET ADNI	AD vs. NOR	resubs.		1.000 [0.000]	1.000 [0.000]	1.000 [0.000]
		MVN	402	0.994 [0.003]	0.988 [0.006]	1.000 [0.007]
		KDE	402	0.811 [0.014]	0.791 [0.021]	0.832 [0.028]
		MVN	40	0.906 [0.014]	0.882 [0.020]	0.930 [0.034]
		KDE	40	0.752 [0.018]	0.736 [0.026]	0.768 [0.030]
	MCI vs. NOR	resubs.		0.999 [0.001]	0.998 [0.002]	1.000 [0.001]
		MVN	402	1.000 [0.000]	1.000 [0.000]	1.000 [0.000]
		KDE	402	0.823 [0.018]	0.843 [0.024]	0.801 [0.033]
		MVN	40	0.722 [0.021]	0.863 [0.024]	0.580 [0.143]
		KDE	40	0.692 [0.015]	0.829 [0.023]	0.556 [0.139]
	MCI vs. AD	resubs.		1.000 [0.000]	1.000 [0.000]	1.000 [0.000]
		MVN	402	1.000 [0.000]	1.000 [0.000]	1.000 [0.000]
		KDE	402	0.820 [0.014]	0.799 [0.022]	0.841 [0.030]
		MVN	40	0.775 [0.025]	0.615 [0.047]	0.934 [0.163]
		KDE	40	0.706 [0.011]	0.573 [0.011]	0.840 [0.135]
DAT PPMI	PD vs. NOR	resubs.		1.000 [0.000]	1.000 [0.000]	1.000 [0.000]
		MVN	268	0.998 [0.002]	0.997 [0.003]	1.000 [0.003]
		KDE	268	0.848 [0.013]	0.809 [0.021]	0.887 [0.043]
		MVN	24	0.966 [0.012]	0.961 [0.018]	0.971 [0.015]
		KDE	24	0.854 [0.024]	0.839 [0.035]	0.869 [0.035]

When using all available components (*L* = 402 for ADNI and *L* = 268 for PPMI) we observe that the performance loss of the MVN estimator is almost null, probably due to the aforementioned overfitting. Conversely, the KDE model produces more different images, increasing the prediction error up to a 0.2 under all scenarios in ADNI and 0.15 in PPMI.

However, this situation changes completely when using the optimal *L* in the MVN synthesis. In this case, the performance loss is higher in all cases, demonstrating that the images are different from the original training set, and less concentrated than when using higher *L*s. Moreover, whereas the KDE achieved similar performance under all scenarios (with all *L*s), the MVN with optimal *L* replicates the baseline behavior found at Table 1; that is, a much higher discrimination ability in AD vs. NOR than when comparing to the prodromal state MCI. This is even true under the two MCI scenarios: the accuracy is higher under the MCI vs. AD scenario (0.775) than in the MCI vs. NOR (0.722), the same that occurred in the original dataset (0.702 and 0.698, respectively).

## 4. Discussion

In this work, we propose an brain image synthesis algorithm that analyses a dataset, extracts its most relevant characteristics and then generates new images that share the same properties. The algorithm is based on PCA, which defines a new common space for each dataset (the “eigenbrain” space), in which the individual images are represented as points. There, it models the distribution of these points, using them to construct a generative model in the eigenbrain space. After generating new samples in the eigenbrain space, these can be inversely transform to the image space, producing new samples of the same population (see Figure 1 for a graphical representation of the procedure).

The use of PCA for feature extraction neuroimaging in components is widely documented (Zhu et al., 1994; Markiewicz et al., 2009; Illán et al., 2011; Khedher et al., 2015; Martinez-Murcia et al., 2016). The PCA components model different orthogonal sources of variance in the original data. In AD, these components, or eigenbrains, represent features that have been associated with the progression of the disease. The contribution of each eigenbrain (or the coordinates of each subject in the eigenbrain space) has been proven to effectively model the advance of the disease in many works. PCA has also been used to build up a reference of the sources of variance in a generative model in Zhu et al. (1994), using a different simulation strategy. Therefore, it was the optimal tool, well-known and with proven utility, to be used in this work.

**Figure 8:**
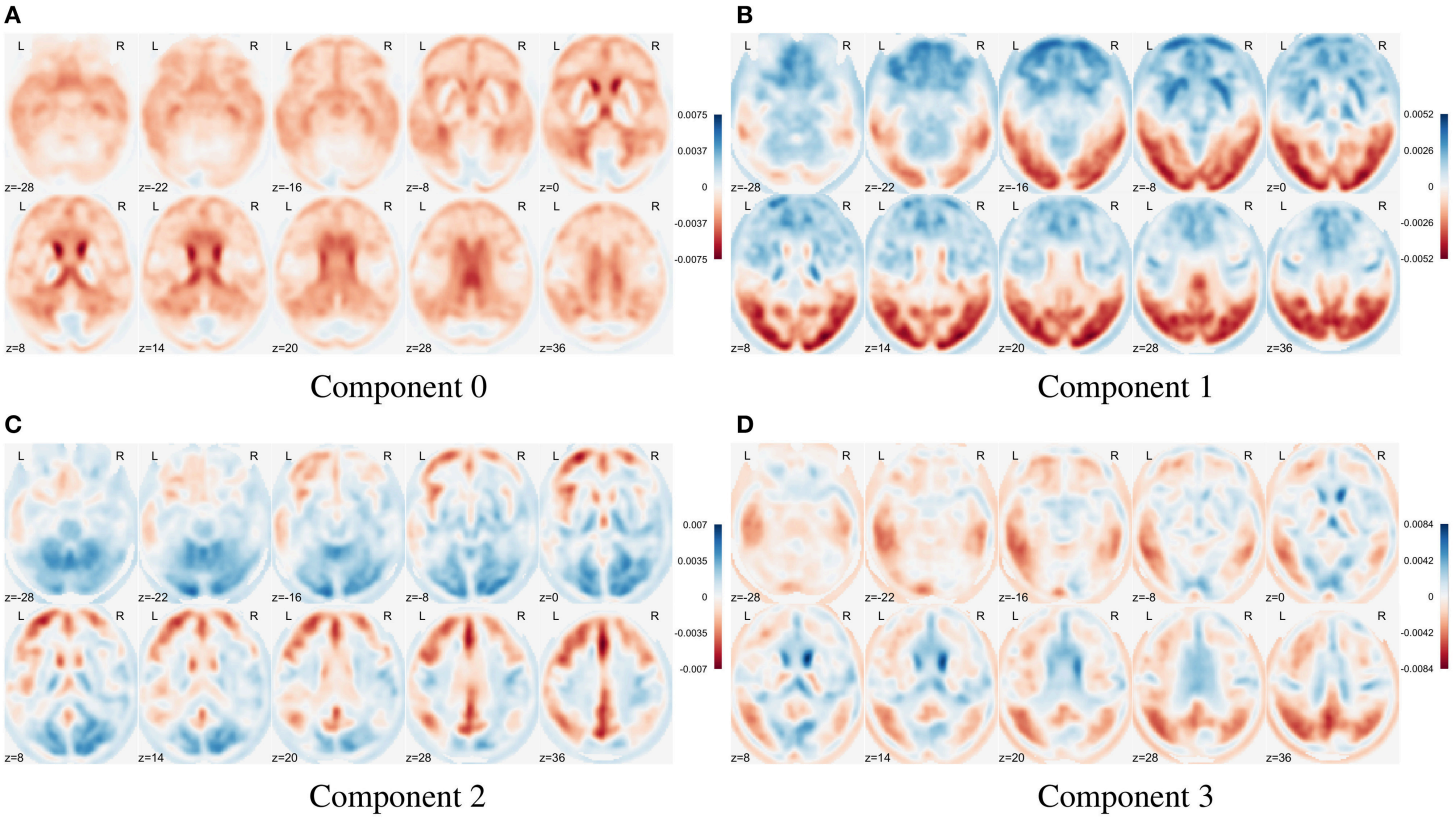
Illustration of the first four eigenbrains (components 0 to 3) of the PET ADNI dataset.

In Figure 8 we show the first four eigenbrains (zero-centered) for the ADNI-PET dataset. The positive/negative contributions of each eigenbrain are shown in blue/red color, respectively. Each component accounts for different variability terms in the original dataset, for example, a negative contribution at the cingulate gyri, precunei, and areas around the thalamus in Component 0 (Stoeckel et al., 2004; Illán et al., 2011), contrast between the anterior and posterior part of the brain in Component 1 and so on. Component 2 accounts for uptake differences at the angular gyrus and precuneus that have been linked to AD (see SPM analysis at section 3.1.1), also present in Component 3. These features prove that the computed eigenbrains are representative of independent structures and activities that together can positively influence the synthesis of new brain images via a correct parametrization and estimation of the component scores.

The distribution of the samples in the eigenbrain space was modeled using two estimation methods: a Multivariate Normal Distribution (MVN) and the Kernel Density Estimation (KDE) via diffusion. An accurate estimation of the distribution of the scores belonging to a certain class is paramount to obtain a reliable brain image synthesis.

**Figure 9:**
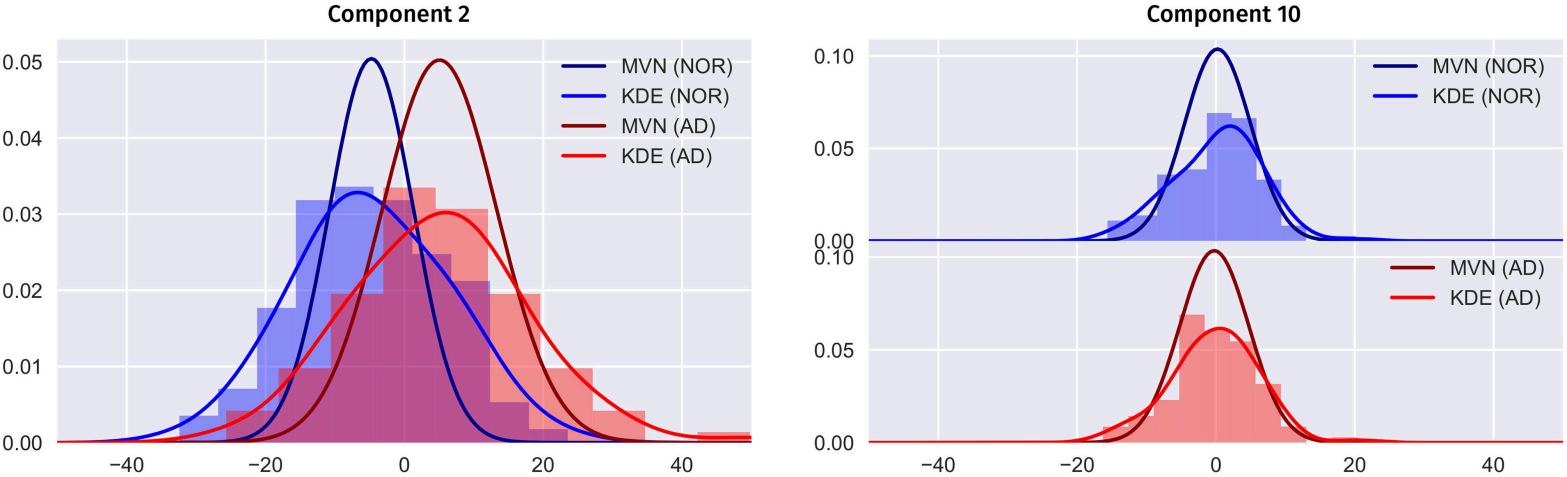
Comparison between the MVN and KDE PDF estimation methods for the component 2 and 10 in the PET ADNI dataset (AD and NOR groups for simplicity), setting the histogram as reference.

In Figure 9 we compare the two PDF estimation methods in two relevant eigenbrains: 2 and 10 (only AD and NOR groups are shown for simplicity), setting the original classes histogram as reference. Note that, since the MVN is multivariate in nature, we have projected the 2nd and 10th components to a single component for a estimated model of *L* = *K* components to obtain a clearer visualization.

Two major features are shown in these figures: class separability and quality of the modeling. As it can be seen, a large proportion of the variability contained in component 2 correspond to class differences (see also Figure 8 and the positive weights of AD-related brain regions). These class differences are higher in the MVN model with *L* = 403 (more concentrated classes) than in the KDE model. Furthermore, the KDE model adapts better to the empirical distribution, as it can be seen in component 10.

On the other hand, the scores distribution may not contain class differences, as in component 10. However, in this case, the distribution of scores in both classes is asymmetric, with longer tails and less gaussian than component 2. So, it is easier to assume that the KDE modeling will also perform better in this case.

Nevertheless, we cannot assume that the PCA components are statistically independent, and therefore, the KDE per-component model is deprecating substantial information. The multivariate nature of the MVN does consider these possible dependences, which are then included in the model. This makes it more accurate but at the same time more prone to overfitting.

**Figure 10:**
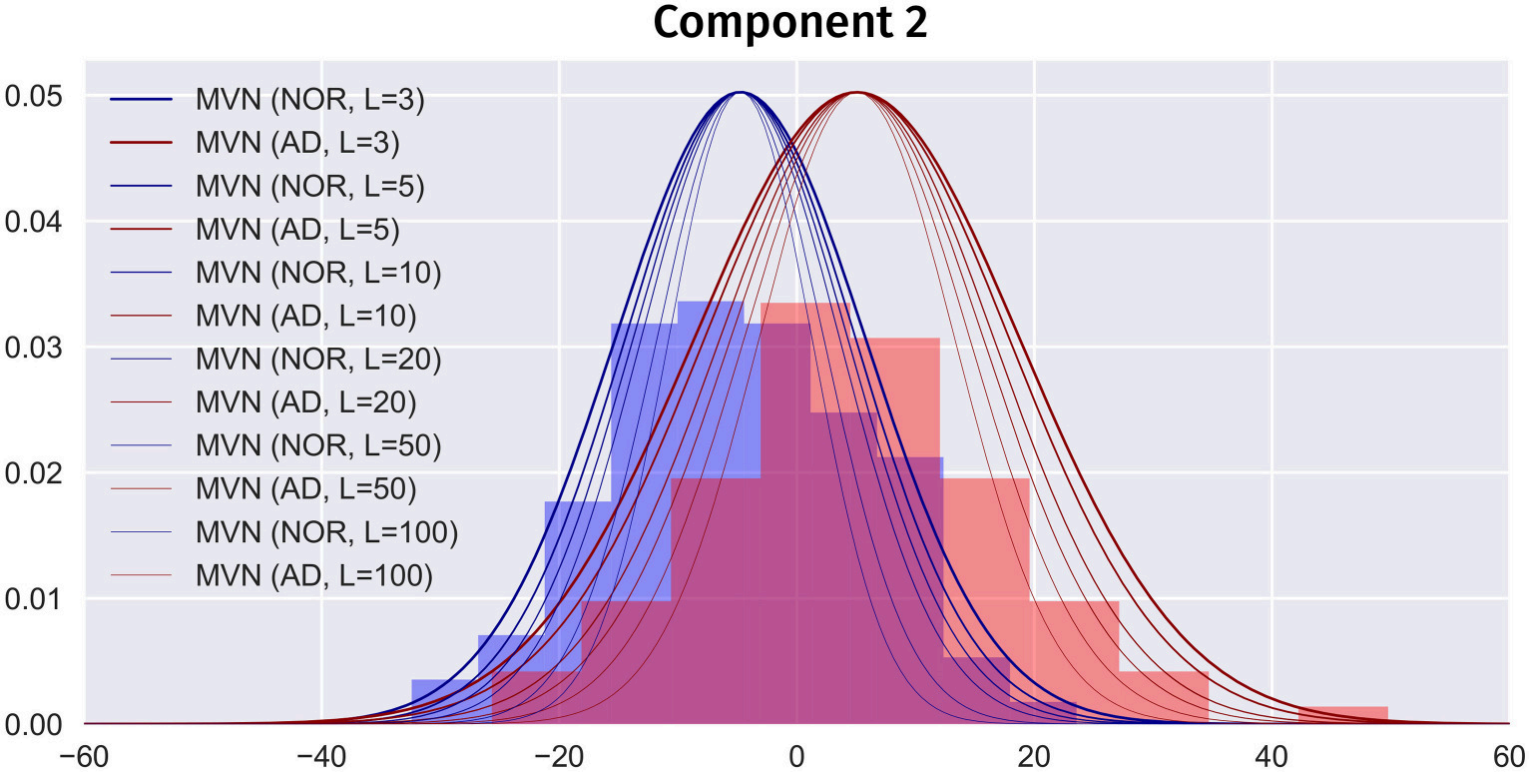
Partial PDF modeling of component 2 (AD and NOR classes) using the MVN estimator with different *L*s. PDFs scaled for comparison.

Overfitting in the MVN model means that, when *L* → ∞, the model would only produce the average image of each class. So, choosing an optimum *L* has a strong impact on the simulated images. A higher *L* would contain more high frequency information, but it also overfits the model, producing images more similar to the average. On the other hand, small *L*s will lead to more overlapped classes, more similar to the real world, but less detail. Figure 10 shows how increasing *L* affects the class overlapping, reducing the variability and eventually leading to images that are very close to each other. In this work, we carefully chose *L* = 40 for ADNI and *L* = 24 for PPMI by selecting the highest number of components that obtained similar performance to the original dataset. A significantly higher performance might be considered overfitting. Other approaches to select an optimum *L* such as the Variance of Reconstruction Error (VRE) proposed in Mnassri et al. (2010) or the modified Bayesian model selection criterion of Kazianka and Pilz (2016) might be considered in the future.

**Figure 11:**
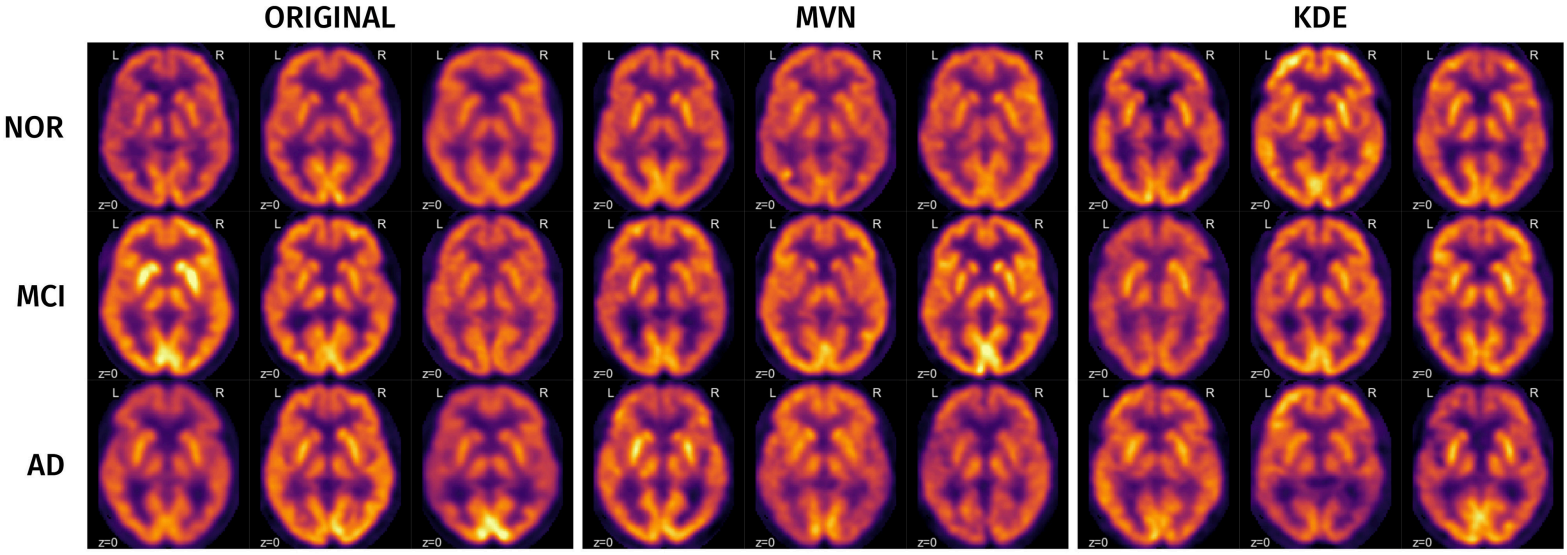
Examples of some original and synthetic subjects from the ADNI-PET dataset.

**Figure 12:**
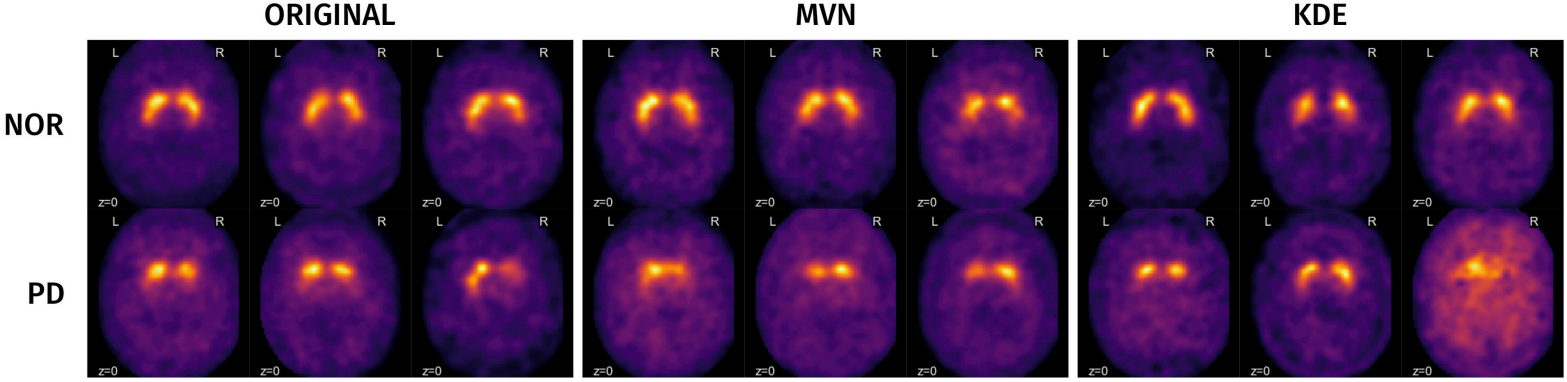
Examples of some original and synthetic subjects from the PPMI dataset.

A visual analysis of the synthetic images in Figures 11, 12 reveals that the synthetic images preserve similar characteristics of the original datasets. For example, it is easy to appreciate differences in glucose metabolism in Figure 11 typically associated with AD, such as a smaller activity at the temporal lobe or a less homogeneous distribution of the radiopharmaceutical in the gray matter (Stoeckel et al., 2004; Illán et al., 2011). In the synthetic DaTSCAN images (Figure 12) differences in shape and intensity of the striatum, and bilateral differences (Lozano et al., 2010; Towey et al., 2011; Illán et al., 2012; Martínez-Murcia et al., 2014) can also be noticed, using both MVN and KDE modeling, although variability of the PD class is perhaps better modeled in KDE. This is again a probable case of overfitting.

When analyzing the particularities of the MVN and KDE modeling under a classification analysis, we have already demonstrated that MVN tends heavily to overfitting when the number of components is high (*L* = *K*), but it considers all conditional probabilities in the estimation. For its part, the KDE modeling is more robust to overfitting, mainly due to its univariate nature (per-component modeling), keeping a relevant prediction ability over real-world images, at the same time that its dependence on the original dataset is reduced. However, when an optimum *L* is chosen, the MVN model resembles more the original dataset performance in both the generalization ability (E1.3) and the dependence of the synthetic images on the original dataset (E2).

Each PDF estimation method has advantages and disadvantages. The KDE modeling works “out of the box,” producing more heterogeneous classes and images. On the other hand, the MVN modeling requires more fine-tuning of the *L* parameter, but it preserves conditional probabilities that may add relevant information to the synthetic images. New multidimensional PDF models, such as a multivariate KDE estimation or alpha-stable (heavy-tailed) modeling of the non-gaussian components, could also be used to preserve these conditional probabilities and improve each component modeling.

Still, evaluation and data availability are currently major bottlenecks for assessing validity and comparing machine learning approaches in neuroimaging, especially with the increasingly popular deep learning approaches. Testing on large samples of dynamically generated images that belong to the same population as the original (as in E2) can produce more realistic performance estimates, leading to a standardization of the evaluation of CAD systems that provides an idea of their generalizing capacity.

Our methodology provides functional brain images that could be drawn from the same population as the original dataset; images that can effectively predict real world samples at the same time that they remain independent from the database used in the modeling. Compared to other widely used data augmentation procedures, such as affine or elastic deformations, it is a more advanced paradigm that can simulate functional patterns associated with a given disease, which could increase the generalization ability of our models. This application is the main purpose of this paper, but apart from this there exist many application possibilities yet to be explored, e.g., using the synthetic images in clinical training of future professionals, or in standardized automatic evaluation procedures.

## 5. Conclusions

In this work, we have proposed a novel brain synthesis algorithm that could be used, among other things, in standardizing evaluation of CAD systems or as a data augmentation procedure. The algorithm consists of an analysis of a existing dataset using Principal Component Analysis, building a space defined by these principal components, or eigenbrains. In this space, we have modeled the spatial distribution of each class, a model from which we can derive new coordinates in the eigenbrain space that can be projected back into the original image space.

We have tested the algorithm in two well-known databases: one FDG-PET database from the Alzheimer's Disease Neuroimaging Initiative (ADNI), and a DaTSCAN SPECT database from the Parkinson's Progression Markers Initiative (PPMI). A visual analysis of the synthetic images revealed that they visually resemble the originals, sharing functional patterns that have been associated with Alzheimer's Disease and Parkinson's Disease in the literature. A Statistical Parametric Mapping analysis revealed similar regions in both the original and the synthetic datasets when studying the significant differences between classes.

We tested different features of the synthetic datasets under three experiments, aimed to prove their ability on detecting real-world image patterns and quantifying their dependence on the original dataset and the number of components used in the modeling. When comparing the two PDF estimation procedures, we found that the Multivariate Normal distribution (MVN) was more accurate, but also more affected by overfitting, whereas the synthesis using Kernel Density Estimation (KDE) produced more overlapped classes at any number of components considered, probably due to missing information about conditional probabilities. Our system, regardless of the PDF estimation, proved to be a useful tool for generating synthetic images that could be used for data augmentation, standardization of CAD system evaluation and even educational purposes.

## Author contributions

Conception or design of the work: FM-M, II, JG, and JR. Data collection: II, JR, JG, and DS-G. Data analysis and interpretation: FM, JG, and JR. Drafting of the article: FM-M, JG, and JR. Critical revision of the article: FS, JG, JR, II, FS, DC-B, and DS-G. Major revision of the article: FM-M, JR, and JG.

### Conflict of interest statement

The authors declare that the research was conducted in the absence of any commercial or financial relationships that could be construed as a potential conflict of interest.

## Supplementary material

Supplementary figures of the SPM analysis of all scenarios and datasets, including the original montages of SPM12 and the glass brain visualization.
