# Supplementary figures and images for: Functional Brain Imaging Synthesis Based on Image Decomposition and Kernel Modeling: Application to Neurodegenerative Diseases
Source: Front Neuroinform. 2017 Nov 14;11:65. doi: 10.3389/fninf.2017.00065 (PMC5694626; doi:10.3389/fninf.2017.00065)

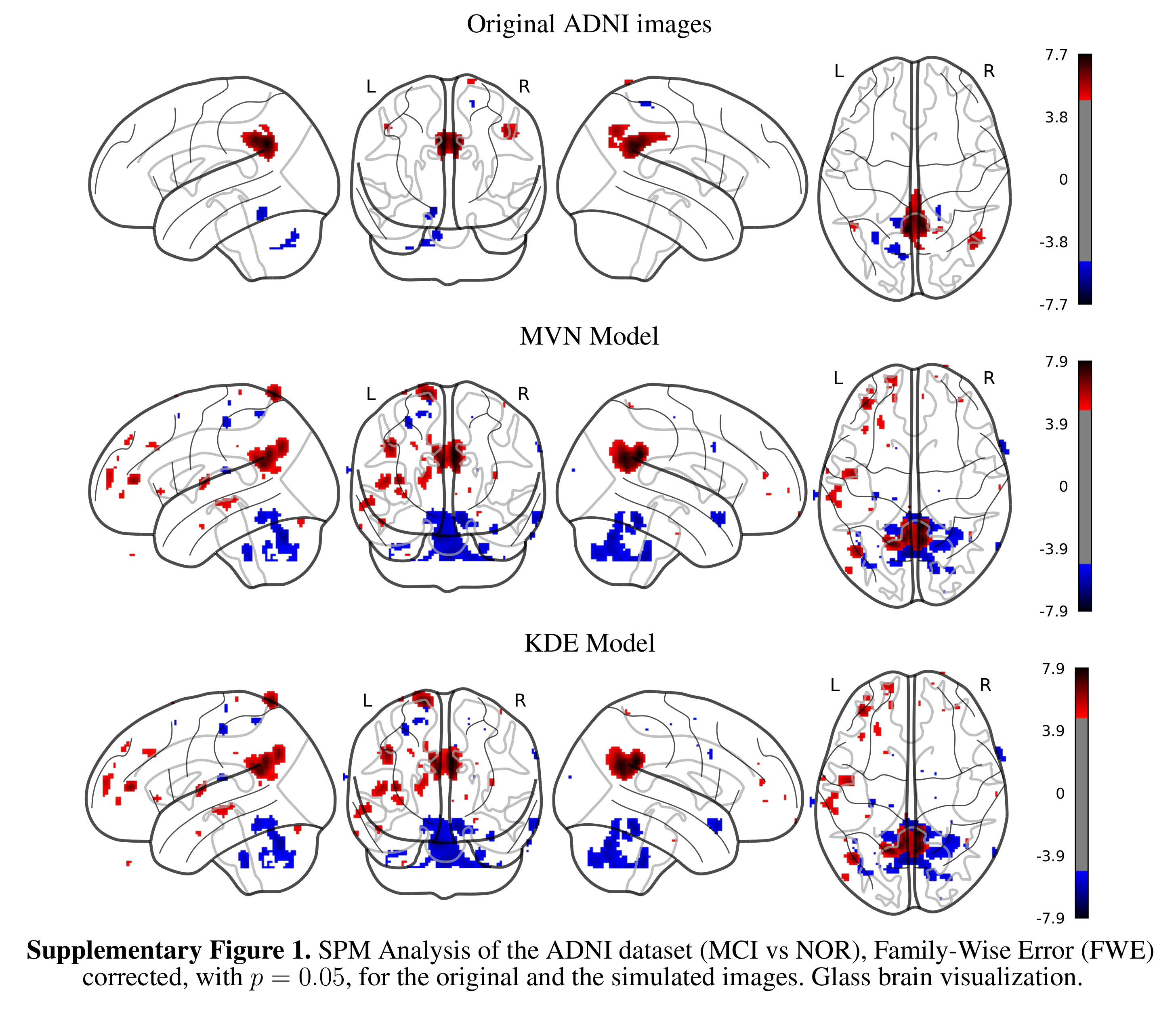

Supplement: Supplementary file 1 [file Image1.TIF]

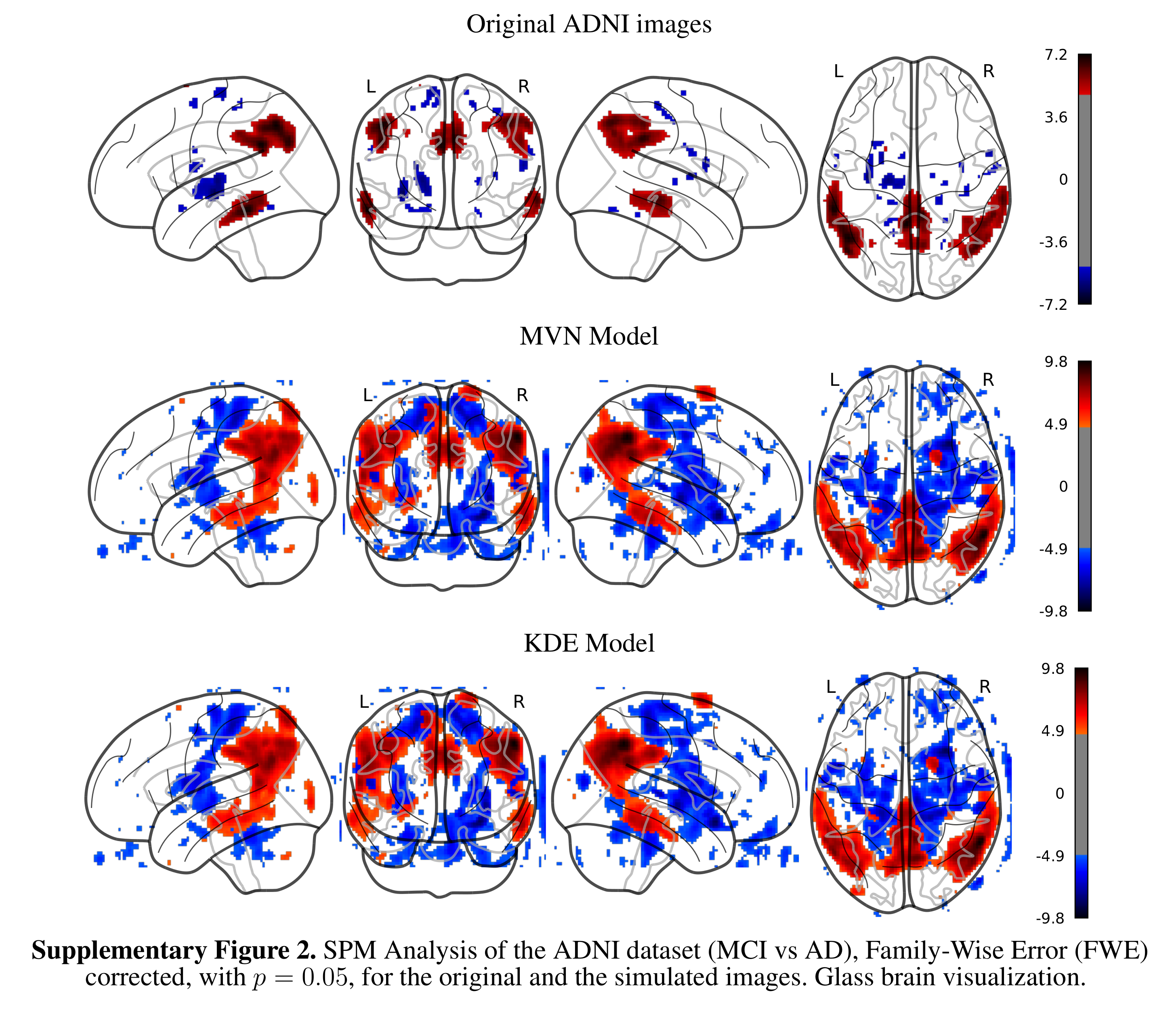

Supplement: Supplementary file 2 [file Image2.TIF]
